# The Bacterial Microbiome in the Small Intestine of Hooded Seals (*Cystophora cristata*)

**DOI:** 10.3390/microorganisms8111664

**Published:** 2020-10-27

**Authors:** Mario Acquarone, Alejandro Salgado-Flores, Monica Alterskjær Sundset

**Affiliations:** Department of Arctic and Marine Biology, UiT—The Arctic University of Norway, 9037 Tromsø, Norway; alejandro.f.salgado@uit.no (A.S.-F.); monica.a.sundset@uit.no (M.A.S.)

**Keywords:** 16S rRNA, metabarcoding, gut, bacteria, arctic, seal

## Abstract

Arctic hooded seals (*Cystophora cristata*) are monogastric carnivores that go through extreme fasting and re-feeding in early life. They are born isolated on sea ice; suckle high-fat milk for four days and may then fast for up to one month before they start hunting and feeding on small prey (fish and crustaceans). Previous studies of the gut microbiota in pinnipeds have focused on the large intestine, while little data exist on the small intestinal microbiota. In this study, the bacterial microbiome in the proximal and distal small intestine of four captive two-year old seals (two males and two females) fed herring (*Clupea harengus*) was sampled post-mortem and characterized using 16S rRNA metabarcoding from the V1–V3 hypervariable region of the 16S ribosomal RNA (rRNA) genes. The seals were originally born in the wild and taken into human care at the end of the suckling period. Molecular-based analysis using Illumina Hiseq resulted in 569,910 16S rRNA sequences from the four seals (both sampling sites together). Taxonomical classification applying a naive Bayesian algorithm gave 412 Operational Taxonomic Units (OTUs). Firmicutes was the major phylum across samples (Proximal (P): 90.5% of total sequences, on average; Distal (D): 94.5%), followed by Actinobacteria (P: 7%; D: 0.3%) and Proteobacteria (P: 1.7%; D: 1.9%). Bacterial spp. belonging to the Clostridium (P: 54.1%; D: 41.6%) and SMB53 (P: 15.3%; D: 21.5%) constituted the major genera in both the proximal and distal small intestine. Furthermore, comparison with hindgut and fecal samples from geographically diverse marine mammals highlighted similarities in the microbiome between our seals and those sharing similar aquatic environments. This study has provided a first reliable glimpse of the bacterial microbiota in the small intestine microbiome of hooded seals.

## 1. Introduction

Hooded seals (*Cystophora cristata*) are deep-diving, long-distance swimmers that occupy vast ocean areas throughout the North Atlantic and adjacent Arctic marine waters [1,2,3]. They spend most of the year at sea presumably foraging except during the breeding and molting periods that are spent in ice-covered waters off the coast of Northeast Canada and Greenland [3,4,5,6,7]. Hooded seal new-born pups undergo extreme nutritional transitions during their first month of life. In four days they almost double their body weight [8] suckling milk with up to 60% fat [9] after which the mother abandons them on the ice. Even though the pups are ingesting large quantities of fat, their digestive organs (stomach, small and large intestines, and the pancreas) are neither particularly large at birth nor do these organs gain in weight or length at unusual speed [10]. The 4-week post-weaning fasting period, during which the pups presumably ingest only snow and seawater [11] ends when the pups begins to hunt and feed on small prey [12,13]. The fast results in weight loss/nutrient depletion, with liver and spleen decreasing in weight by about 70% so that they are actually lighter one month postpartum than at birth [10].

Seals are monogastric carnivores with simple hindguts (a rudimentary cecum and a short simple colon) [14,15]. The stomach is cylindroid with a sharp pyloric bend, it weighed between 395 and 2080 g and had a pH ranging between 1.2 and 7.1 in 40 hooded seals examined (body mass 37–2015 kg) [15]. The concentration of pepsin, an endopeptidase key for protein breakdown, was found to be significantly higher in hooded seals that had been eating a mixed diet of fish and crustaceans (146 µg mL^−1^) compared to only crustaceans (50 µg mL^−1^) [15].

The relative length of the pinniped small intestine varies considerably. Some species have significantly longer small intestines than those in terrestrial carnivores of similar size [14,16]. In hooded seals, the length of the small intestine is 13× body length, while the small intestines of the northern elephant seals (*Mirounga angustirostris*) and southern elephant seals (*Mirounga leonina*) average 25× body length and only 4.8× body length in the Ross seal (*Ommatophoca rossii*) [16,17,18]. The hypothesis that the relatively long small intestine of some seal species evolved to compensate for long periods of reduced or even abolished blood perfusion to the intestine during diving [19] was later rejected, as no significant correlation was found between relative intestinal length and diving ability [16]. Instead, the area of the small intestine was significantly related to body length/size [16].

Little is known about the hooded seal diet; however, stomach content analyses indicate that adult seals feed on large prey. This diet may vary considerably depending of the location where seals happened to be sampled and can include large cod, halibut, and redfish [20]. A diet study using quantitative fatty acid signature analysis of hooded seals sampled along the Northeastern coast of Newfoundland and Southern Labrador indicated a diet of amphipods, Atlantic argentine, capelin, euphausiids and redfish [21] highlighting that the hooded seal diet is variable. Transitions from feeding to fasting, and from fasting to feeding, pose severe challenges to the digestive system of vertebrates [22]. Symbiosis between mammals and their gut microbiome is important for the extraction of energy and nutrients from food and influences both immune response and brain development. The gastrointestinal tract is colonized during birth and then by maternal, social, and environmental contact. The composition of the adult gut microbiome depends on initial colonization, food chemistry and intake, and hereditary aspects such as host-genetics. Several studies have described the gut microbiota in pinnipeds [23,24], which is mostly dominated by a ‘core’ of a few bacterial phyla [25,26]. Nonetheless, these studies were focused on the microbiota from the large intestine of the digestive tract. There are few data available on the microbiome from the small intestine despite the essential role of this section of the gut in carbohydrate and lipid metabolism [27]. Here we present the first study on the bacterial microbiome in the small intestine in the hooded seal—a mammalian model for extreme fasting and re-feeding.

## 2. Materials and Methods

### 2.1. Animals and Sampling

We sampled and characterized the bacterial microbiome from the proximal and distal small intestine (contents) of four healthy adult hooded seals (age 2 years), two males (81.5 kg and 86 kg) and two females (88 kg and 103 kg).

The seals had originally been born in the wild and taken in human care after weaning between the 26th and the 28th March 2012 (by permit from the Danish Foreign Ministry and the Greenland authorities). They were transported fasted, by ship from Greenland to Tromsø, Norway and maintained in two 40,000 L sea water pools in the approved animal holding facilities at the Department of Arctic and Marine Biology of UiT—The Arctic University of Norway. During the period in human care the seals were fed freshly thawed, human food grade frozen herring (*Clupea harengus*) integrated daily by marine animal dietary supplement (Sea Tabs ^®^ MA, Pacific Research Labs Inc., PO Box 675890, Rancho Santa Fe, CA 92067, USA). The animals were euthanized by bleeding in isoflurane anesthesia and were also sampled extensively as part of other research projects unrelated to this study (permit no. 5399 (reference 2013/87412-119) issued by the National Animal Research Authority of Norway 3rd June 2013).

All seals received routine antiparasitic treatment with ivermectin (10 mg sub cutaneous injection) upon arrival at the facility on 3rd April. An oral antiparasitic treatment was also administered for three days starting on 22nd June and consisted on a total dose of 250 mg of Panacur.

One of the females was treated with antibiotics for an infection in the jaw (enrofloxacin 300 mg oral dose daily between 4th and 12th December 2012 and again between 5th and 14th March 2013). One of the males also suffered from an infection of the jaw and received a daily intra-muscular (i.m.) injection with enrofloxacin (400 mg) 31st July–11th August 2013 followed up by a daily oral dose (300 mg) between 12th and 16th August. The same animal was treated with a daily oral dose of enrofloxacin (400 mg) between 23rd and 30th December and between 7th and 14th February 2014 for a new infection of the jaw. This last treatment was administered 12 days before sampling.

For this study, the abdominal cavity of the freshly euthanized animal was immediately opened and the intestinal system, sectioned at the end of the duodenum and before the colon, was removed from the carcass. The contents of approximately 2 m at each end of the removed intestinal tract were manually squeezed out of the gut and into 50 mL centrifuge tubes. The samples were immediately frozen and kept at −40 °C, until analysis.

### 2.2. DNA Extraction

DNA extraction was based on the protocol of the Repeated Bead Beating plus Column (RBB+C) Method developed by Yu and Morrison [28]. DNA quantification was done with NanoDrop 2000c spectrophotometer and solutions were stored at −20 °C until PCR amplification.

### 2.3. Sequencing

PCR amplifications for Bacteria were performed with the bacterial primer set 27F (5′-AGAGTTTGATCCTGGCTCAG -3′and 519R (5′-GWATTACCGCGGCKGCTG -3′) [29,30], giving a 500-nt size amplicon product targeting the V1-V3 hypervariable region of the 16S ribosomal RNA (rRNA) genes. PCR reactions were run as described in [31]. Sample products were then pooled in equimolar amounts, checked in a 1% agarose gel electrophoresis, and excised and purified from gel using a NucleoSpin Gel and PCR Clean-up kit (Macherey-Nagel, Düren, Germany). The resulting DNA was stored at −20 °C until sequencing. PCR amplicons were sequenced with Illumina MiSeq at the MrDNA company (Shallowater, TX, USA).

### 2.4. Sequence Processing

Sequences for 16S rRNA genes from both microbial groups were analyzed using the Quantitative Insights Into Microbial Ecology (QIIME) (v. 1.9.0) pipeline [32]. First, Illumina forward and reverse fast reads were merged using the join_paired_ends.py script. The resulting merged reads were quality checked and discarded when: length <200; homopolymers runs exceeded 6 nts; average quality scores fall below 25; and the presence of primer mismatches. Operational taxonomic units (OTUs) clusters were produced with QIIME-incorporated USEARCH software at a 97% similarity threshold, applying a word length of 64. Chimeric sequences were flagged and automatically discarded using the UCHIME [33] tool in QIIME.

### 2.5. Sequence Analysis

Representative sequences from each OTU, i.e., the most abundance sequence assigned to a specific cluster, were aligned against a reference database (Greengenes v .13_5; May 2013 release) applying a version of the Near Sequence Alignment Tool (NAST) algorithm based on Python [34], implementing a 75% cut-off and 150 nt word length. The Ribosomal Database Project RDP-classified tool [35] integrated in QIIME was used for taxonomy identification of the chimera-free OTUs, based on a Naïve-Bayesian algorithm on 8-km at an 80% cut-off, against the RDP-II project database as reference. Alpha diversity indicators evaluating intra-group species richness (chao1), evenness (Shannon) and overall diversity (Simpson) were analyzed using the alpha_diversity.py script in QIIME from randomly subsampled dataset.

To facilitate direct comparisons across studies, the raw dataset was also processed with the open-source pipeline DADA2 (v1.16.0; May 2020 release) [36]. DADA2 entails a different approach compared to traditional OTU-based clustering by processing exact sequences as Amplicon Sequence Variants (ASVs). The lack of clustering and use of exact sequences allows for higher reproducibility when compared to ASVs covering a comparable target region. The dataset was processed following the standard recommended pipeline parameters (https://benjjneb.github.io/dada2/tutorial.html v1.16; May 2020). The resulting sequence table including all the filtered, trimmed, merged and chimera-free ASVs was made available (Table S1).

### 2.6. Statistical Analysis

Considering the limited sample size for the current study (*n* = 4), it may not be sufficient to conduct robust statistical comparisons. Consequently, we have made a special emphasis on presenting a qualitative report of the bacterial communities dwelling the proximal and distal part of the small intestine in the captive hooded seals as a proxy for potential future, broader-scope assessments.

## 3. Results

### 3.1. Bar Chart Plot

**Figure 1:**
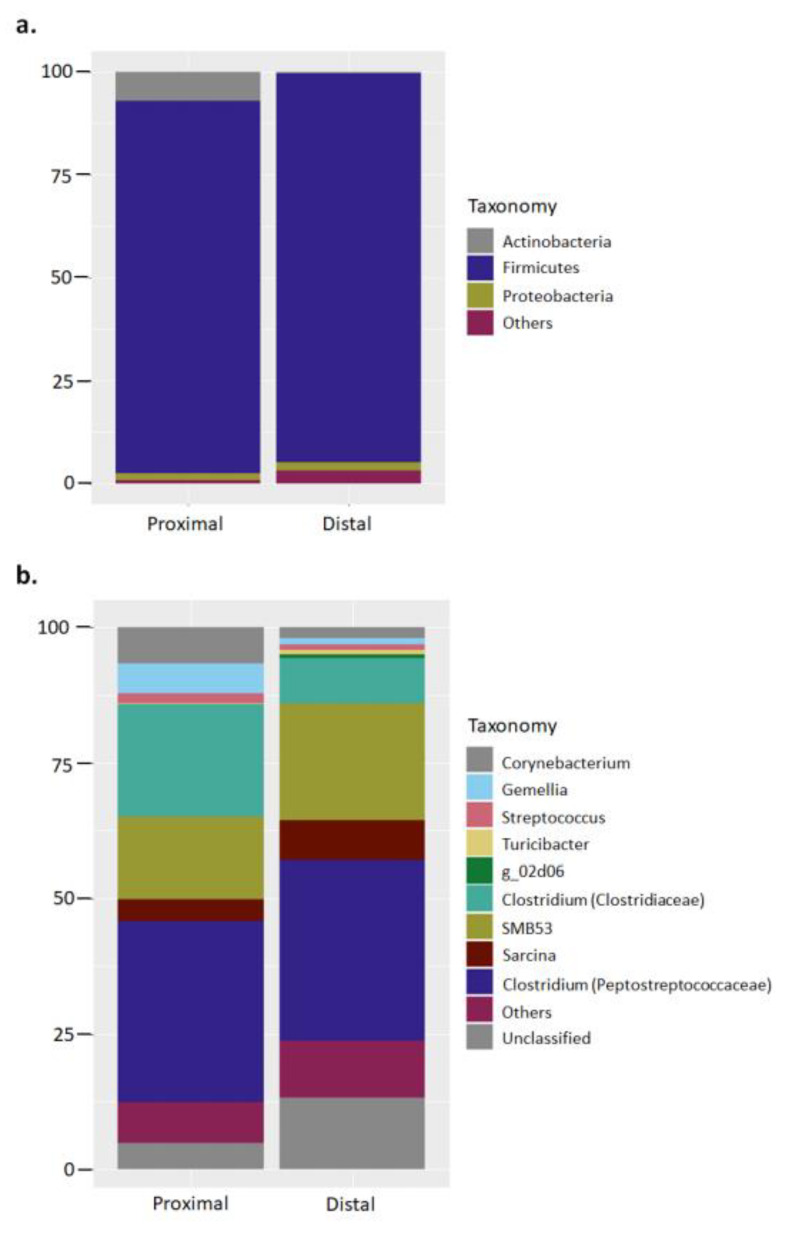
Major bacterial phyla and genus in the proximal and distal small intestine of captive hooded seals. The bar charts represent the relative abundance of the total 16S rRNA sequence taxonomically classified at (**a**) phylum and (**b**) genus level. Classification was performed with partial sequences of the bacterial 16S rRNA gene against the RDP-II database using RDP classifier tool.

A total 1,081,347 bacterial 16S rRNA sequences were produced from small intestine proximal (*n* = 4) and distal (*n* = 4) samples of hooded seals. Quality checked samples resulted in a range between 57,381 and 72,161 sequences per sample, with an average length of 405 bases for downstream analysis. Based upon a 97% similarity criterion the sequences were clustered in 412 chimera-free OTUs. Taxonomical classification at phylum level showed a microbiota dominated by the phylum Firmicutes in both proximal and distal small intestine samples (Proximal: 90.5 ± 12.2% of total sequences, on average; Distal: 94.5 ± 4%). Actinobacteria (Proximal: 7 ± 12.7%; Distal: 0.4 ± 0.3%) and Proteobacteria (Proximal: 1.7 ± 1.5%; Distal: 1.9 ± 2.3%) constituted the remaining major phyla (Figure 1a; Figure S1). The standard deviation in the relative abundance at phylum level ranged from 0.9% to 12.7% for the proximal sample group, and 0.3% to 4% for the distal sample group.

Classification at genus level showed bacterial phylotypes assigned to Clostridium from the families Peptostreptococcaceae (Proximal: 33.4 ± 32.7%; Distal: 33.3 ± 26.8%) and Clostridiaceae (Proximal: 20.7 ± 32.1%; Distal: 8.3 ± 7.2%) as the major genera, followed by genus SMB53 (Proximal: 15.3 ± 14.5%; Distal: 21.5 ± 15.2%), and unclassified genera from the family Peptostreptococcaceae (Proximal: 4.8 ± 6.96%; Distal: 13.3 ± 11.5%) (Figure 1b). The standard deviation in the relative abundance at genus level ranged from 0.1% to 32.7% for the proximal sample group, and 1.4% to 26.8% for the distal sample group.

**Figure 2:**
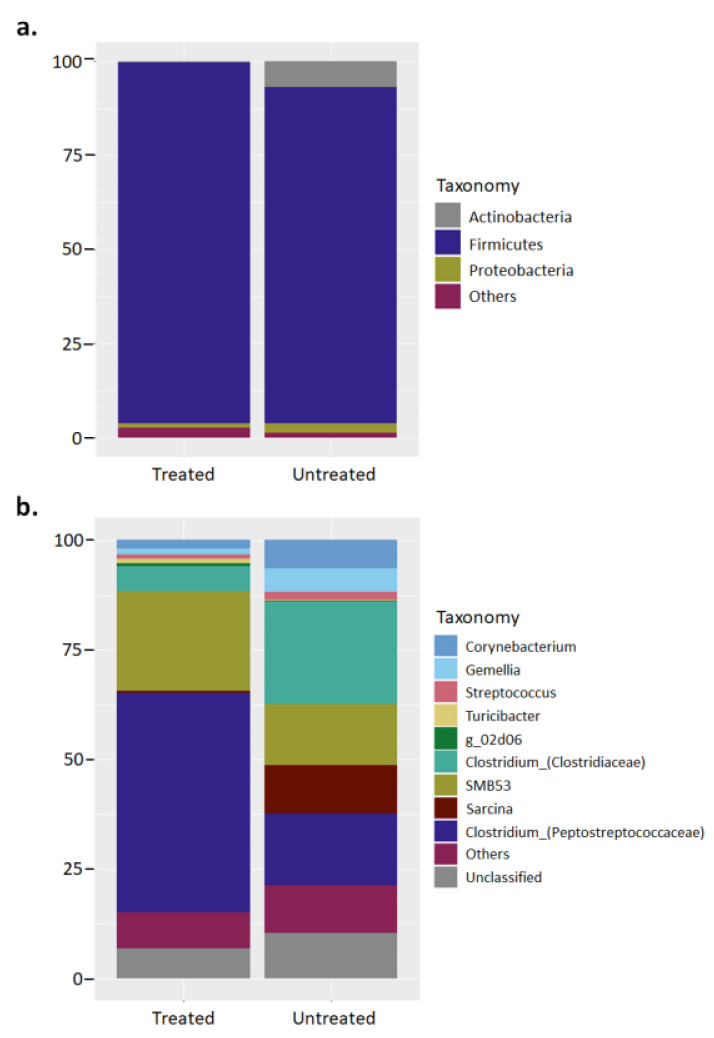
Major bacterial phyla and genus in the proximal and distal small intestine of captive hooded seals depending on whether they received antibiotic treatment or not. The bar charts represent the relative abundance of the total 16S rRNA sequence taxonomically classified at (**a**) phylum and (**b**) genus level. Classification was performed with partial sequences of the bacterial 16S rRNA gene against the RDP-II database using RDP classifier tool.

Some of the studied seals were provided occasional antibiotic treatment (enrofloxacin) throughout the captive period. Considering the widely reported impact posed by antibiotics on the intestinal microbiota [37], taxonomical results were also presented separating those seals receiving antibiotics and not receiving antibiotics to assess for potential differences (Figure 2). As observed, classification at phylum level is also represented by a microbiota dominated by the phylum Firmicutes (Treated: 95.6 ± 4.1%; Untreated: 89.3 ± 11.4%) (Figure 2a). Classification at genus level showed seals treated with antibiotics to be dominated by member of the genus Clostridium from the family Peptostreptococcaceae (50.2 ± 22.5%) and family Clostridiaceae (22.9 ± 14%) (Figure 2b). Likewise, the bacterial microbiota from untreated seals was also dominated by the genus Clostridium from the family Peptostreptococcaceae (16.5 ± 23.1%) and family Clostridiaceae (23.5 ± 31.1%); however, other genera were also importantly represented such as SBM53 (13.9 ± 14.7) and Sarcina (11 ± 13.6). Interestingly, “unclassified” bacteria constituted a comparable relative proportion of the total microbiota in both treated (7.7 ± 10.5) and untreated seals (10.4 ± 10.7) (Figure 2).

### 3.2. Group-Based Diversity Tests

Intra-group (alpha) diversity indicators for samples grouped based on sampling site (i.e., proximal or distal small intestine) showed comparable values for species richness (chao1), evenness (Shannon), and overall diversity (Simpson’s) (Figure S1a–d).

Similar parameters were interrogated when samples were grouped based on antibiotic treatment (Figure S3a–d). Overall, alpha diversity parameters were comparable for every individual metric between both groups of samples; however, Simpson diversity index was on average lower in samples from seals undergoing antibiotic treatment (Figure S3c).

### 3.3. Comparison with the Microbiota from Other Marine Mammals

To assess the findings presented here from a broader perspective, we compared them with the results described from other marine mammals (Table 1). Due to the lack of information of the microbiota in the small intestine in general, and in particular for marine mammals, all the additional data were obtained from studies using cecal or fecal samples. In addition, some of the studies applied different sequencing platforms to the one used in the current study (Illumina HiSeq) or hybridization-based techniques, aspects that may potentially pose bias to the results. The majority of the listed species possessed a gut microbiota dominated by the phylum Firmicutes, similar to that reported in the current study (Figure 1a). Only a few studies reported animals whose gut microbiota was dominated by other microbial phyla apart from Firmicutes. It was interesting to observe that the fecal microbiota in wild hooded seals was dominated by Bacteroidetes (68% relative sequence abundance), in contrast to the results from our captive seals (no presence of Bacteroidetes). In addition, other phocids such as harbor seals presented a more balanced microbiota where Firmicutes and Bacteroidetes were the dominant phyla, both in captive and wild seals. Actinobacteria (5%) and Proteobacteria (2%), which were present at a very low relative abundance in the current study, constituted an important fraction of the microbiota in some species such as wild and captive Australian sea lion, wild leopard seals, and porpoises from China (Table 1). Fusobacterium was also largely present in some marine mammals such as southern elephant seals and captive leopard seals, but this phylum was not detected in the small intestine of the captive hooded seals.

## 4. Discussion

### 4.1. Homogenous Bacterial Microbiome Along the Small Intestine of the Hooded Seals

The microbiome of the small and the large intestine in mammals differs fundamentally, but only limited information is available about the microbiota of the small intestine, despite its relevance to many physiological mechanisms and also pathological states [27]. The small intestine is characterized by a relatively short transit time, and also influx of bile and digestive enzymes that creates harsh conditions not favorable for a bacterial growth. The small intestinal microbiome adapts rapidly to changes in availability of nutrients and metabolizes simple carbohydrates for community maintenance [43]. Studies from human samples reported differences in the bacterial communities throughout sections of the small intestine, with a higher overall bacterial density and increased presence of anaerobic bacteria toward the distal ileum where transit is slower [27]. However, a major concept shown across studies is that the microbiota of the small intestine is less diverse than that of the large intestine although more dynamic. In the current study diversity indicators assessing species richness and evenness gave similar results for samples from the distal and proximal small intestine of hooded seals (Figure S1a–d). A cross-sectional analysis of the bacterial microbiota in the digestive tract of a Brazilian ruminant showed dissimilar communities between the duodenum and ileum, but in multi-dimensional analysis of those samples clustered together when compared with samples from other gastrointestinal tract (GIT) sections [44]. Dissimilar bacterial profiles were also found between samples from the small intestine and cecum in swine with different fatness [45]. Such results indicated the presence of a comparable microbiota along the small intestine, but different compared to other GIT compartments. Furthermore, the same microbial groups were found to dominate the proximal and distal part of the small intestine in the studied captive hooded seals at phyla and genus level (Figure 1a,b), which suggests the existence of a homogeneous microbiota across this organ. The nature of the ingested food, consisting mostly of fats and proteins that are mostly digested and absorbed in the small intestine, together with a short retention time, would leave little substrate for fermentation in these carnivorous animals. Thus, these aspects would lead to the growth of a similar microbiota between the proximal and distal parts.

### 4.2. Comparing the Small Intestine Microbiome of the Hooded Seal to That of Other Marine Mammals

A previous study describing the colonic microbiota in wild hooded seals showed dominance by the phylum Bacteroidetes followed by Firmicutes [23]. In contrast, the current study presented Firmicutes as the dominant bacterial taxa, and the absence of Bacteroidetes-related phylotypes (Figure 1a). One factor that may potentially influence bacterial diversity is the living conditions, i.e., captivity versus free-ranging state. For instance, the samples used in the current study were collected from captive seals, and captivity has been discussed to largely influence the gut microbiota composition in other marine mammals such as leopard seals (*Hydrurga leptonix*), where increased relative abundance of Firmicutes-related bacteria was described in the captive animals [26]. A presumably less varied diet given to the captive leopard seals was accounted for the main cause driving to such differences (see discussion in [26]). Likewise, wild hooded seals combine a diet including several fish species, squid, and some invertebrates (crustaceans) [21], which overall constitutes a richer diet than that fed to our captive seals (herring and dietary supplements; see Methods). In addition, other factors directly associated with a captive lifestyle, such as the administration of antibiotics, and physiological alterations (e.g., hormonal production), may also trigger such differences [25,46]. Nonetheless, as previously discussed, the part of the digestive tract chosen for sampling (i.e., colon vs small intestine) would pose a stronger effect on the composition of the bacterial microbiota and would account for most of the differences observed between wild and captive hooded seals. 

Clostridium constituted the dominant genus in the small intestine of the studied captive hooded seals (Figure 1b). The ubiquity of this genus in the digestive tract of several other marine mammals, from Arctic and Antarctic latitudes, has made it to be considered to be part of a putative ‘phocid seals core microbiota’, together with several other bacterial genera [24,25]. In principle, such a shared microbiota would be passed from mother to pups, and it would be involved in several physiological aspects, from host-immunity, maturation of the gut tissue, and, the breakdown of milk components during the 2-month period after birth [25]. The lack of data on the small intestine microbiota across marine mammals gives us no possibility to associate the presence of *Clostridium* spp. with a putative core microbiota. Nonetheless, a potential link between an increase in the relative abundance of bacterial members belonging to the class Clostridia and the consumption of diets high in fat have been reported [27], which may hint a role played by these bacteria in fat metabolism in this intestinal section.

The genus SMB53 also constituted a substantial fraction of the microbiota in the small intestine of our captive hooded seals (Figure 1b). SMB53 is a poorly studied genus belonging to the class Clostridia, which has mostly been reported in captive hosts such as birds, pigs, and obese laboratory mice [45,47,48]. SMB53 was also present in free-ranging carnivores consuming a high-fat, high-protein diet, but it constituted a small fraction of the microbiota [49]. In addition to the current study, the only record of this genus in the small intestine was found in farmed pigs with high body fatness, whose microbiome was enriched in inflammation-related genes speculated to trigger increased fat adiposity [45]. Whether the presence of this genus in our captive seals is directly linked to the consumption of a high-fat, high-protein diet or altered by antibiotics (which captive animals are normally provided) remains to be elucidated until more information on its physiological features is reported.

### 4.3. Potential Effect of Antimicrobial and Antiparasitic Treatment on the Gut Microbiota

The ingestion of antibiotics has been reported to pose an impact on the gut microbiota [37]. For instance, broad-spectrum antibiotics may drop the overall community diversity, an effect that may last months or even years [37]. In the current study, two of the four captive seals received occasional antibiotic treatment (enrofloxacin) due to an infection in the jaw (see Methods). Enrofloxacin is a fluoroquinolone antibiotic commonly used for animals; some studies described a decrease in overall diversity in the gut microbiota of laboratory mice, with a particular emphasis in members from the family Bacteroidaceae [50]. Likewise, in this study, a reduction in microbial richness (Simpson index) was observed in samples from captive hooded seals that were administered this antibiotic (Figure S3c), which showed dominance by one particular group belonging to the genus Clostridium (family Peptostreptococcaceae) (Figure 2b). Although the use of antibiotics has been commonly associated with an increase in members of the phylum Bacteroidetes over Firmicutes [37], some pathogenic bacteria such as *Clostridium difficile* (Firmicutes) may also be favored after antibiotic treatment [37]. As indicated in the Methods section, statistical analysis was not addressed in the current study due to limitation in sample size, which makes it impossible to test the differences in bacterial taxonomy between treated and untreated hooded seals. Nonetheless, in light of the result for both groups of seals (treated vs. untreated with antibiotics), a direct link between the use of antibiotics and dominance by Firmicutes may be discarded.

Knowledge of the potential effects exerted by the use of antiparasitic drugs on the gut microbiome has been investigated less than for antibiotics, with some studies performed in human subjects indicating a drop in overall diversity as well as alterations in some individual microbial groups [51]. In the current study, all captive hooded seals underwent an intitial treatment with ivermectin (a macrocyclic lactone) and Panacur (fenbendazole), broad-spectrum antiparatic drugs categorized as anthelmintics (see Methods). Research on the effect posed by each specific drug on the gut microbiota is limited and mostly shown a lack of effect in community-wide diversity [52,53]. Instead, certain positive effect was observed at individual level to bacteria within the families Enterobacteriaceae and Lachnospiraceae [52]. Based on this information and considering that antiparasitic treatment was administered exclusively upon arrival to the animal facilities at UiT, almost two years prior to sampling, we may suggest a negligible effect posed by the administration of either drug on the small intestine microbiota in the captive hooded seals in this study.

### 4.4. Importance of the Gut Microbiota in Food Digestion in Carnivores

Although the small intestine is the main site for the complex physiological processes of protein and lipid digestion and absorption, its associated microbiota has been substantially neglected compared to other GIT regions, e.g., the large intestine. Still, some studies have described its composition and reported the potential role assigned to this microbial consortium on lipid metabolism, mainly by increasing lipid absorption via enteroendocrine signaling in the proximal small intestine (duodenum-jejunum) [54,55]. For instance, when germ-free mice were transplanted with the small intestinal microbiota of mice conditioned on a high-fat diet, lipid absorption was significantly increased regardless the fat content in the diet [56]. Some microbial phyla (e.g., Firmicutes) have been associated with increased fat absorption and metabolic disorders such as obesity [49], although the extent of the effect is a matter of debate [57]. All the marine mammals used for the interspecies comparison were carnivores ingesting generally a diet rich in fat and protein (Table 1), with most of them presenting a gut microbiota dominated by Firmicutes bacteria. Our captive hooded seals were mostly fed herring, a foodstuff rich in protein and polyunsaturated fatty acids (PUFAs) such as omega-3 [58]. It has been reported that ingestion of omega-3 dietary supplements is linked with an increase in butyrate-producing bacteria belonging to the phylum Firmicutes [59]. Altogether, it may be speculated that dominance by Firmicutes-related bacteria in the small intestine of captive hooded seals may be related to the ingestion of a fatty acid-rich carnivore diet, which in turn, may play a role in easing lipid metabolism and energy uptake. New experiments targeting to unveil the genetic information (metagenomics) must be conducted to elucidate the functional potential in fat metabolism exerted by the small intestine microbiota in hooded seals or other carnivore marine mammals.

## 5. Conclusions

Despite the increasing body of knowledge regarding the gut microbiota of marine mammals, there is still little information available on their small intestine microbes for both captive and wild individuals. To date, the current study is the first endeavor to unveil the bacterial constituents found in the small intestine of captive hooded seals originally captured in the pack ice off the coast of East Greenland. No significant differences were observed between the bacteria found in the proximal and distal parts of the small intestine, indicating a seemingly homogenous microbiota throughout the small intestine. We suggest that the diet of fatty fish, which is mostly digested in the small intestine, would leave little material for subsequent bacterial degradation at distal sections and, therefore, rendered a similar microbiota at both ends of the small intestine. Dominance by bacterial groups previously associated with individuals feeding on high-energy diets, in some instances also related to metabolic disorders (e.g., obesity), may indicate a small intestine populated by microbes adapted to metabolize foodstuffs rich in fat and protein. Such microbiota would help maximize the degradation and energy retrieval from the diet. In addition, a broad comparison with the microbiota reported from several other marine mammals allowed us to identify the presence of microbial groups in our captive seals that have been hypothesized to belong to a putative phocid core microbiota. Nonetheless, any potential conclusion drawn by direct comparisons should be considered with care until more information on the small intestine microbiome can be obtained from other marine mammals. Further analyses of the small intestine microbiome at a functional level (i.e., metagenomics/metatranscriptomics) would help understand the role played by such microbes in physiological functions related to lipid absorption and energy metabolism, relevant to comprehend the digestive physiology in hooded seals.

## Figures and Tables

**Table 1:** Intestinal microbiota composition of carnivore marine mammals from Arctic and Antarctic latitudes. The results were presented per host animal species, living conditions (wild or captive), and sampling origin. Taxonomy was indicated at phylum level considering the total relative abundance. An indication on the applied methodology by which the results were obtained was also included.

Species	Latin Name	Location—Diet	Samples	Bacterial Community	Method	Reference
**Hooded seal**	*Cystophora cristata*	Wild (Greenland Sea)—Halibut, herring, cod, squid, crustaceans	Colon	Bacteroidetes (68%) Firmicutes (22%) Proteobacteria (9%)	BigDye—Sanger sequencing	[23]
**Harbor seal**	*Phoca vitulina*	Wild (Ringvassøy, Troms)—saithe, cod, herring, sculpin	Colon	Firmicutes (50%) Bacteroidetes (49%)	BigDye—Sanger sequencing	[23]
**Gray seal**	*Halichoerus grypus*	Wild (Ringvassøy, Troms)—Cod, saithe, herring, sandeel, catfish	Colon	Firmicutes (76%) Bacteroidetes (24%)	BigDye—Sanger sequencing	[23]
**Harbor seals**	*Phoca vitulina*	Captive – herring, sprat, crustaceans	Feces	Firmicutes (33 ± 11%) Bacteroidetes (27 ± 7%) Fusobacteria (26 ± 4%) Proteobacteria (13 ± 5%)	454 pyrosequencing	[24]
**Southern elephant seals**	*Mirounga leonina*	Wild (Western Antarctica)—squid, fish	Rectal swab	Firmicutes (42 ± 10%) Bacteroidetes (22 ± 5%) Fusobacteria (20 ± 8) Proteobacteria (16 ± 8%)	454 pyrosequencing	[26]
**Leopard seals**	*Hydrurga leptonyx*	Wild (Western Antarctica)—seals, krill, penguins, fish/captive (Taronga zoo)—fish	Rectal swab	Firmicutes (45 ± 13%) Proteobacteria (33 ± 12%) Fusobacteria (14 ± 8%) Firmicutes (60 ± 32%) Fusobacteria (34 ± 13%)	454 pyrosequencing	[26]
**Australian fur seals**	*Arctocephalus pusillus doriferus*	Wild (Kanowna island, Australia)—fish and cephalops	Feces	Firmicutes (67%) Bacteroidetes (15%) Actinobacteria (3%)	FISH	[38]
**California sea lion**	*Zalophus californianus*	Captive—fed fish and squid	Rectal swab	Bacteroidetes (40 ± 21%) Firmicutes (29 ± 20%) Fusobacteria (25 ± 9)	Sanger sequencing	[39]
**Australian sea lion**	*Neophoca cinerea*	Wild—benthic fish, squid, lobster, small crustacean)/captive—frozen fish	Feces	Firmicutes (74 ± 28%) Proteobacteria (10 ± 20%) Bacteroidetes (9 ± 16%) Firmicutes (58 ± 33%) Proteobacteria (30 ± 35.9%) Bacteroidetes (5 ± 8.6%)	Illumina MiSeq sequencing	[40]
**South American fur seals**	*Arctocephalus australis*	Wild dead animal (Southern coast of Brazil)—fish, cephalopods, crustaceans	Feces	Firmicutes (89 ± 6%) Proteobacteria (6 ± 6%) Actinobacteria (3 ± 2%)	Ion Torrent	[41]
**Subantarctic fur seals**	*Arctocephalus tropicalis*	Wild dead animal (Southern coast of Brazil)—fish, cephalopods, crustaceans, rock hoper penguins	Feces	Firmicutes (84 ± 6%) Actinobacteria (11 ± 3%)	Ion Torrent	[41]
**Yangtze porpoise**	*Neophocaena phocaenoides asiaeorientalis*	Wild (Jianxi, China)—fish, crustaceans, cephalopods	Feces	Firmicutes (51.3%) Tenericutes (17.9%) Proteobacteria (15.4%) Actinobacteria (7.7%)	Sanger sequencing	[42]
**Hooded seals**	*Cystophora cristata*	Captive—herring and diet supplements	Small intestine	Firmicutes (93 ± 9%) Actinobacteria (4 ± 9%) Proteobacteria (2 ± 3%)	Illumina HiSeq sequencing	Current study

## Data Availability

The sequence reads obtained from 16S amplicon sequencing and shotgun metagenomics are available at the Sequence Read Archive (SRA) database under the BioProject identifier PRJNA665267 (SAMN16249586-SAMN16249593).

## Supplementary Materials

The following are available online at https://www.mdpi.com/2076-2607/8/11/1664/s1, Figure S1: Major bacterial groups in the small intestine of treated and untreated with antibiotics captive hooded seals. The bar charts represent the relative abundance of the total 16S rRNA sequence taxonomically classified at (**a**) class, (**b**) order, and (**c**) family level. Classification was performed with partial sequences of the bacterial 16S rRNA gene against the RDP-II database using RDP classifier tool. Figure S2: Boxplot intra-group microbial diversity indicators from proximal and distal small intestine samples. Mean alpha diversity values for: (**a**) species richness (Chao1); (**b**) evenness (Shannon index); (**c**) overall diversity (Simpson); and (**d**) total observed species. Figure S3: Boxplot intra-group microbial diversity indicators from the small intestine of treated and untreated with antibiotics captive hooded seals. Mean alpha diversity values for: (**a**) species richness (Chao1); (**b**) evenness (Shannon index); (**c**) overall diversity (Simpson); and (**d**) total observed species. Table S1: Amplicon Sequence Variants (ASVs) from the proximal and distal parts of the small intestine of Hooded seals. ASVs were obtained from raw sequencing dataset and processed as indicated in Materials and Methods (2.5. Sequence Analysis).
